# A Pilot Study of Inhaled CO Therapy in Neonatal Hypoxia-Ischemia: Carboxyhemoglobin Concentrations and Brain Volumes

**DOI:** 10.3389/fped.2018.00120

**Published:** 2018-05-01

**Authors:** Martha Douglas-Escobar, Monique Mendes, Candace Rossignol, Nikolay Bliznyuk, Ariana Faraji, Abdullah S. Ahmad, Sylvain Doré, Michael D. Weiss

**Affiliations:** ^1^Department of Pediatrics, University of California, San Francisco, San Francisco, CA, United States; ^2^Department of Anesthesiology, Center for Translational Research in Neurodegenerative, McKnight Brain Institutive, University of Florida, Gainesville, FL, United States; ^3^Department of Pediatrics, University of Florida, Gainesville, FL, United States; ^4^Department of Agricultural and Biological Egineering, University of Florida, Gainesville, FL, United States; ^5^Department of Neurology, Center for Translational Research in Neurodegenerative, McKnight Brain Institutive, University of Florida, Gainesville, FL, United States; ^6^Department of Psychiatry, Center for Translational Research in Neurodegenerative, McKnight Brain Institutive, University of Florida, Gainesville, FL, United States; ^7^Department of Neuroscience, Center for Translational Research in Neurodegenerative, McKnight Brain Institutive, University of Florida, Gainesville, FL, United States

**Keywords:** babies, ischemic stroke, preclinical, therapeutic gas

## Abstract

**Objective:** The objective of this pilot study was to start evaluating the efficacy and the safety (i.e., carboxyhemoglobin concentration of carbon monoxide (CO)) as a putative neuroprotective therapy in neonates.

**Study Design:** Neonatal C57BL/6 mice were exposed to CO at a concentration of either 200 or 250 ppm for a period of 1 h. The pups were then sacrificed at 0, 10, 20, 60, 120, 180, and 240 min after exposure to either concentration of CO, and blood was collected for analysis of carboxyhemoglobin. Following the safety study, 7-day-old pups underwent a unilateral carotid ligation. After recovery, the pups were exposed to a humidified gas mixture of 8% oxygen and 92% nitrogen for 20 min in a hypoxia chamber. One hour after the hypoxia exposure, the pups were randomized to one of two groups: air (HI+A) or carbon monoxide (HI+CO). An inhaled dose of 250 ppm of CO was administered to the pups for 1 h per day for a period of 3 days. At 7 days post-injury, the pups were sacrificed and the brains analyzed for cortical and hippocampal volumes.

**Results:** CO exposure at 200 and 250 ppm produced a peak carboxyhemoglobin concentration of 21.52 ± 1.18% and 27.55 ± 3.58%, respectively. The carboxyhemoglobin concentrations decreased rapidly, reaching control concentrations by 60 min post exposure. At 14 days of age (7 days post injury), the HI+CO (treated with 1 h per day of 250 ppm of CO for 3 days post injury) had significant preservation of the ratio of ipsilateral to contralateral cortex (median 1.07, 25% 0.97, 75% 1.23, *n* = 10) compared the HI+A group (*p* < 0.05).

**Conclusion:** CO exposure of 250 ppm did not reach carboxyhemoglobin concentrations which would induce acute neurologic abnormalities and was effective in preserving cortical volumes following hypoxic-ischemic injury.

## Introduction

Hypoxic-ischemic encephalopathy (HIE) is a serious birth complication due to systemic asphyxia (1). The incidence of HIE ranges from 1 to 8 per 1,000 live births in developed countries and as high as 26 per 1,000 live births in underdeveloped countries (2). Until recently, treatment of HIE consisted of supportive care including respiratory support, treatment of hypotension, careful monitoring of fluid and electrolytes, and treatment of seizures. In the last decade, research has shown that therapeutic hypothermia improves the neurological and neurodevelopmental outcome of a subgroup of infants with moderate HIE (3–6). Therapeutic hypothermia decreases mortality and improves the neurological and neurodevelopmental outcome of up to 53% of treated infants (3, 5–8). Neonates with mild HIE have been excluded from hypothermia trials due to earlier studies which showed that these neonates did not have long-term handicaps (9). However, emerging data has shown that neonates with mild HIE may be at risk for brain injury. Currently, there is not a specific neuroprotective therapy for neonates with mild HIE.

Contrary to the traditional view of carbon monoxide (CO) as a toxic agent (10), CO can be neuroprotective at low-doses (11–13). Exogenous CO has anti-inflammatory, anti-apoptotic and vasodilation effects that are cytoprotective (12–14). *In vitro*, CO preconditioning of neurons prevents apoptosis after induced excitotoxicity and oxidative stress (15). We previously showed that low doses of inhaled CO administered immediately after transient focal ischemia reduced cortical infarct volumes and improved neurological outcomes (16). Our laboratory also discovered that CO regulates the transcriptional factor Nrf2, a key factor in controlling the entire cell antioxidant system through the ARE-Nrf2-Keap1 pathway (17). Although CO has demonstrated great promise in adult animals, there is paucity of knowledge about the potential use of CO as a neuroprotective agent in neonatal animals.

As an initial step in investigating the therapeutic benefit of CO for neuroprotection following mild hypoxic-ischemic brain injury in neonates, we by examined the carboxyhemoglobin over time in neonatal mice exposed to concentration of 200 and 250 ppm of CO exposure for 1 h. These chosen doses were based on animal models of lung injury and our laboratory's results, which show neuroprotection for stroke at these doses (16, 18, 19). Next, a pilot study was performed to test if CO was neuroprotective in a neonatal mouse model of HI. The volumes of cortical and hippocampal injuries were examined in CO-treated HI pups compared with HI air pups and sham controls. We hypothesize that low-dose CO would be safe and preserve brain cortical and hippocampal volumes in a mouse model of neonatal HI.

## Materials and methods

All procedures and anesthetics on the animals were performed in accordance with University of Florida and NIH regulations governing the ethical care and handling of laboratory animals and were approved by the IACUC at the University of Florida.

### Neonatal HI mouse model

The Rice-Vannucci neonatal HI model, which was validated to induce brain injury similar to that seen in neonates with HIE (20, 21). The rat model was modified and validated for use in mice (22) and we have previously established hypoxic-ischemic injury using this model (23–25). Surgical carotid ligation was performed under anesthesia (isoflurane induction 5% and maintenance 2–3%) for an average of 5–10 min. Briefly, the left carotid artery was isolated and ligated in 7-day-old pups. Following the procedure, all pups were allowed to recover for 1 h with the dam. The pups were then placed in a Billups-Rothenburg hypoxic chamber, which was perfused with a humidified gas mixture of 8% oxygen and 92% nitrogen for 20 min. A gel pad (Deltaphase Isothermal Pad, Braintree Scientific Inc., Braintree, MA) was used to ensure the animals maintained a normal temperature during the procedures and during perfusion with the hypoxic gas source. A group of sham-operated animals underwent anesthesia and a surgical incision but did not have their carotid artery ligated. To control for sex and litter variations between litters, each pup from a litter were randomized to one of three groups: Sham operated, hypoxia-ischemia+air (HI+A) or hypoxia-ischemia+carbon monoxide (HI+CO). Due to the randomization, the sex of the pups was not initially recorded. Recording of sex was performed midway through the experiments.

### Temperature monitoring

Prior to the hypoxia exposure, the pups had a baseline temperature taken with a Traceable infrared thermometer (Fisher Scientific, Waltham, MA). Immediately following the hypoxia exposure, the temperature was recorded. In addition, the pups that were exposed to CO had a baseline recording prior to exposure to CO and immediately at the completion of the CO exposure for the pups in the cortical and hippocampal studies. A single temperature measurement from the abdomen was performed prior to, during and after CO exposure.

### Carboxyhemoglobin concentration in neonatal pups

To analyze the carboxyhemoglobin concentration, the 7-day-old neonatal pups were exposed to two inhaled doses of CO at 200 and 250 ppm. These chosen doses were based on animal models of lung injury and our laboratory's preliminary results, which show neuroprotection for stroke at these doses (16, 18, 19). The pups were placed in the hypoxic chamber. Thermoregulation of the pups was obtained using a gel pad (Deltaphase Isothermal Pad, Braintree Scientific Inc., Braintree, MA) during CO exposure. The pups were exposed to the CO for 1 h. A gas analyzer attached to the outflow will strictly monitor the CO levels in the chamber. Following CO exposure, the pups were sacrificed at 0, 10, 20, 60, 120, 180, and 240 min after completion of CO and blood was collected for carboxyhemoglobin analysis.

### Carboxyhemoglobin measurements

An avoximeter was used to measure hemoglobin levels in the blood following CO exposure to evaluate the therapeutic window for CO administration. Carboxyhemoglobin was measured using the Avoximeter 4000, a whole blood CO-oximeter. The animals were exposed to 250 ppm of CO for 1 h while being kept warm with a space gel pad. They were sacrificed at various times after the exposure, and whole blood was collected by cardiac puncture using a heparinized syringe. The syringe, containing the sample, was connected to the cuvette and held at a 45°. The cuvette was filled by gently pressing the syringe plunger until the sample reached the vent patch. The cuvette, with the syringe still attached, was placed in the test chamber. After 10 s the hemoglobin (g/dL), % carboxyhemoglobin, % oxyhemoglobin, and % methemoglobin were displayed. In an effort to reduce animal numbers, the number of mice was decreased for the 250 ppm exposure group compared to the 200 ppm group since the measurements had little standard deviation. In addition, the number of pups was gradually decreased for the later time points since they approached control pup values.

### CO therapy for HI

After the hypoxic exposure, the pups were allowed to recover with the dam for 1 h. The HI pups were then randomized to one of two groups: air (HI+A) or carbon monoxide (HI+CO). An inhaled dose of 200 ppm of CO was administered to the pups 1 h after completion of hypoxia using the Billups-Rothenburg chamber. The pups were thermoregulated using a gel pad during CO exposure. The pups were exposed to the CO for a period of 1 h. A gas analyzer attached to the outflow will strictly monitor the CO levels in the chamber. Seven days post-CO exposure, the pups were deeply anesthetized with 5% isoflurane and perfused with 4% PFA. The brains were then collected for volume analysis.

### Cresyl violet staining

The cerebellum was removed and each brain was completely sectioned using a cryostat at a thickness of 30 μm/section (approximately 120 sections/brain). The fixed frozen sections were mounted on Superfrost Plus slides (Fisher Scientific, Waltham, MA) from −80°C freezer and air-dry overnight at room temperature. The slides were hydrated in 70% ethanol followed by 50% ethanol and finally in distilled H_2_O for 3 min each, and then placed in 0.5% Cresyl Violet acetate (Electron Microscopy Sciences, Hatfield, PA) for 12 min. Each slide was then dehydrated in ascending ethanol and citrasolve, and cover-slipped with permount.

### Volume analysis

A Zeiss axiophot equipped with a Microfire CCD camera (Optronics, Goleta, CA) was used for volume analysis. Real-time images were analyzed using the Stereologer 2000 version 2.1 (Stereology Resource Center, Chester, MD). The injured cortex and hippocampus were identified and outlined on every section. The procedure was then repeated for the uninjured cortex and hippocampus.

### Weights, scoring testing and surface righting

All three groups were weighed on the day of surgery and 3 days after the surgery following the last CO exposure. For each CO exposure, the pups were observed prior to and after. A scoring system was used with Level 1 = unconscious, Level 2 = conscious but weak, Level 3 = standing, Level 4 = alert, nursing well. The pup were placed on their back on a bench pad and held for 5 s. The pups were released and the time it took for them to return to a prone position and the direction of righting were recorded. The test was repeated 3 times. Testing occurred at 24 h post-injury. The CO exposed mice had received 2 doses of CO at the time of testing.

### Statistical analysis

The injured cortex and hippocampus was standardized to the uninjured cortex. All statistical analyses were performed using SAS 9.3 (SAS Institute Inc., Cary, NC) and graphs created using GraphPad Prism (GraphPad Software Inc., La Jolla, CA). One-way ANOVA models were considered for the standardized response data. For each of the two data sets, homogeneity of variance assumption was verified using Bartlett's test (*p*-values greater than 0.5). The results from the normal theory (untransformed) responses were validated nonparametrically using the Kruskal–Wallis test. For the temperature analysis, a paired *t*-test was used to test the treatment effect; every subject serves as their own baseline. All data are represented as mean ± sd. A power analysis was not performed to determine sample size because this was a pilot project.

## Results

### Carbon monoxide exposures

**Figure 1:**
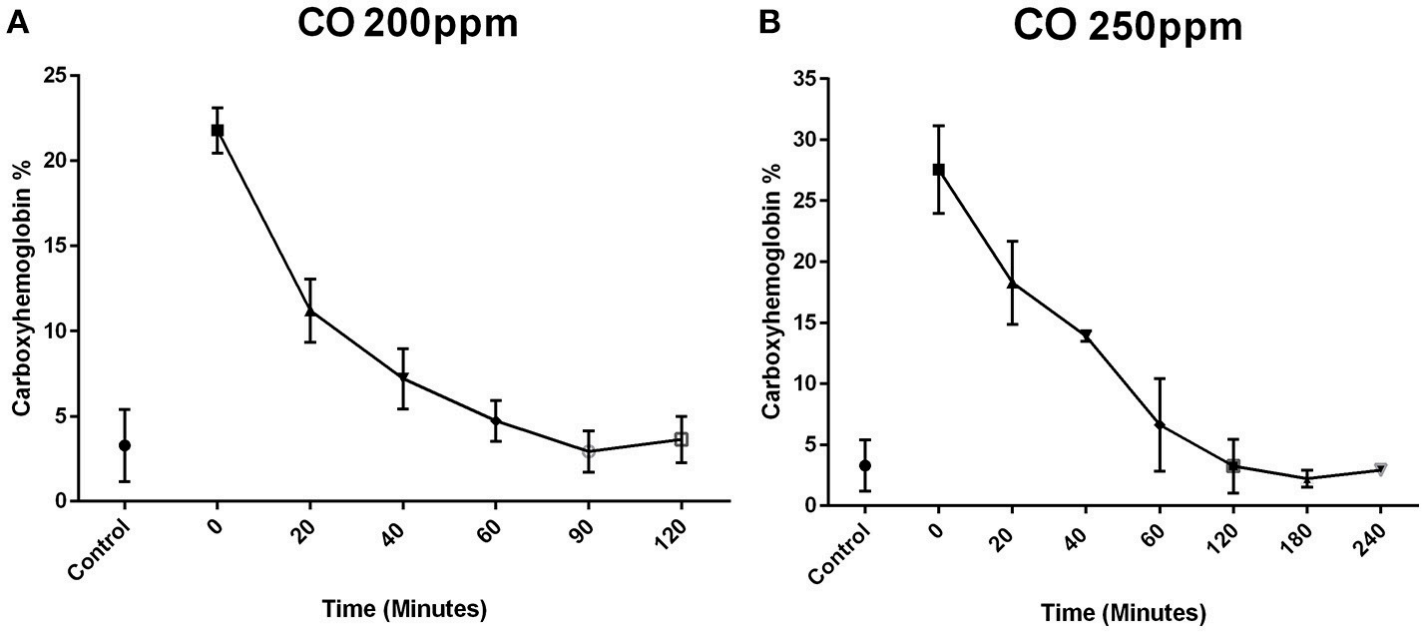
Carboxyhemoblobin levels following exposure to CO in neonates. Mouse pups were exposed to CO for 1 h. Time 0 represents the sampling at the completion of the 1-h exposure. Blood concentrations of CO in mouse pups exposed to CO at 200 ppm **(A)** and 250 ppm **(B)** are shown over time after completion of the infusion. In the mice exposed to 200 ppm, the mean carboxyhemoglobin concentration was 21.5 ± 1.3% (*n* = 6) after the 1-h exposure (time 0). The carboxyhemoglobin concentration decreased to 10.8 ± 1.9% at 20 min (*n* = 6), 8.1 ± 1.8% at 40 min (*n* = 6), 4.7 ± 1.2% at 60 min (*n* = 4), 2.9 ± 1.2% at 90 min, and 3.6 ± 1.4% at 120 min **(A)**. Exposure to 250 ppm of CO produced a higher mean blood carboxyhemoglobin concentration after the 1-h exposure (time 0) of 27.6 ± 3.6% (*n* = 4). The carboxyhemoglobin concentration was 17.4 ± 3.4% at 20 min (*n* = 4), 13.9 ± 0.4% at 40 min (*n* = 2), 6.7 ± 3.8% at 60 min (*n* = 4), 3.2 ± 2.2% at 120 min (*n* = 4), 2.2 ± 0.7% at 180 min (*n* = 3) **(B)**. By 60 min post exposure, the concentrations of carboxyhemoglobin were similar to the control group that was not exposed to CO (control group 3.9 ± 2%).

Exposure of P7 mice to 200 ppm CO for 1 h resulted in a mean carboxyhemoglobin concentration of 21.52% ± 1.18% (*n* = 6) after the 1 h exposure (time 0). The carboxyhemoglobin decreased to 10.83 ± 1.29% at 20 min (*n* = 6), 8.12 ± 1.31% at 40 min (*n* = 6), 4.73% ± 1.20% at 60 min (*n* = 4), 2.93 ± 1.21% at 90 min (*n* = 3), and 3.63 ± 1.36% at 120 min (*n* = 3) (Figure 1A).

Exposure of P7 mice to 250 ppm CO produced a higher mean blood carboxyhemoglobin concentration after the 1-h exposure (time 0) of 27.55 ± 3.58% (*n* = 4). The carboxyhemoglobin concentration was 18.28 ± 3.41% at 20 min (*n* = 4), 13.90 ± 0.42% at 40 min (*n* = 2), 6.62 ± 3.79% at 60 min (*n* = 4), 3.22 ± 2.21% at 120 min (*n* = 4), and 2.20 ± 0.70% at 180 min (*n* = 3) (Figure 1B). By 60 min post exposure, the concentrations of carboxyhemoglobin were similar to the control group which was not exposed to CO control group (3 ± 2%).

Comparing the two dosages of CO, 200 and 250 ppm, there was a significant increase in the concentration of carboxyhemoglobin at 0, 20, and 40 min at a concentration of 250 ppm compared to 200 ppm (*p* < 0.05). There was no difference after 40 min at the other time points examined. The concentrations of carboxyhemoglobion in both the 200 and 250 ppm exposed animals were similar to air control pups at 60 min.

**Figure 2:**
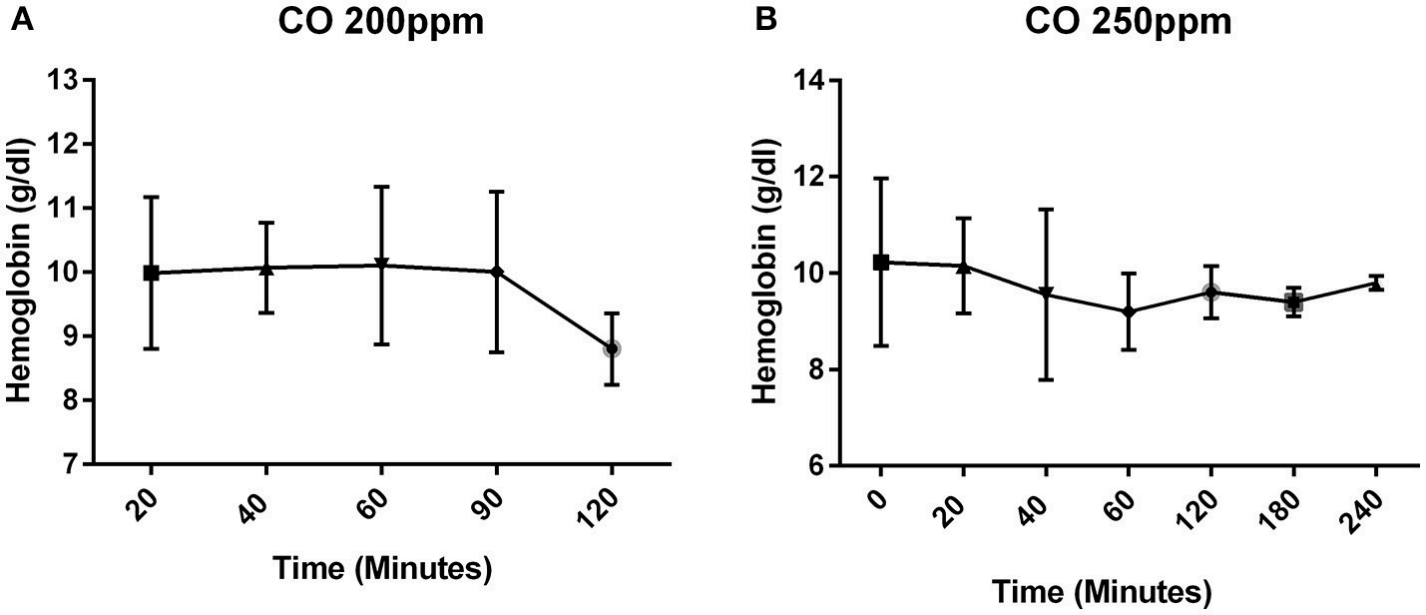
Concentration of hemoglobin over time. The concentration of hemoglobin (g/dl) in mice that received CO at 200 ppm **(A)** and 250 ppm **(B)**.

Since the carboxyhemoglobin is based on a percentage which can be affected by the total hemoglobin, the hemoglobin was measured to verify that there was not a change postnatally during the period of time in which CO was administered. The hemoglobin concentration remained stable postnatally (Figure 2). All pups that were exposed to CO at 200 and 250 ppm survived the exposure.

### Temperatures

The mean (±sd) temperature for the pups that underwent hypoxia (HI+A and HI+CO) were 33.28 ± 1.03°C prior to hypoxia and 33.22 ± 0.68°C immediately following hypoxia (*p* > 0.05). Prior to CO exposure, the mean temperature were 32.72 ± 0.96°C and immediately after 34.7 ± 0.51°C (*p* > 0.05).

### Cortical volumes

**Figure 3:**
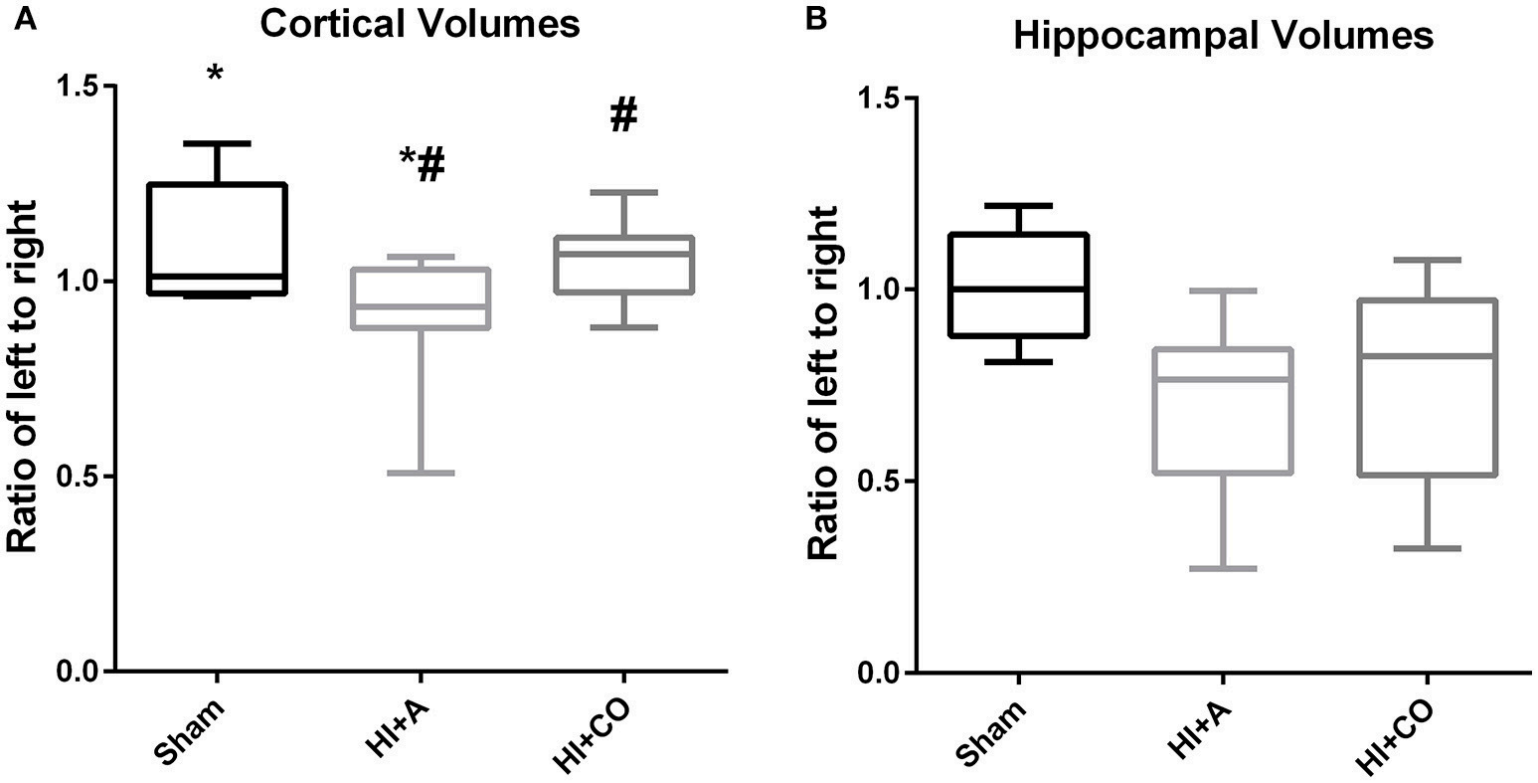
Cortical and hippocampal volumes. At 12 days of age (5 days post injury), the brain region volumes are represented as the ratio of the left (injured)/right (uninjured). **(A)** The cortical volumes of HI+A were decreased compared to sham pups (*n* = 8, ^*^p < 0.05). The HI+CO (treated with 1 h per day of 250 ppm of CO for 3 days post injury) had significant preservation of the ratio of ipsilateral to contralateral cortex compared the HI+A group (*n* = 10, #*p* < 0.05). **(B)** The hippocampal volumes of the HI+A (median 0.76, 25% 0.52, 75% 0.84, *n* = 9) were decreased compared to the sham pups (*n* = 5, *p* < 0.05). The HI+CO (treated with 1 h per day of 250 ppm of CO for 3 days post injury) preservation of the ratio of ipsilateral to contralateral hippocampus was not significantly different from the HI+RA group (*n* = 10).

**Figure 4:**
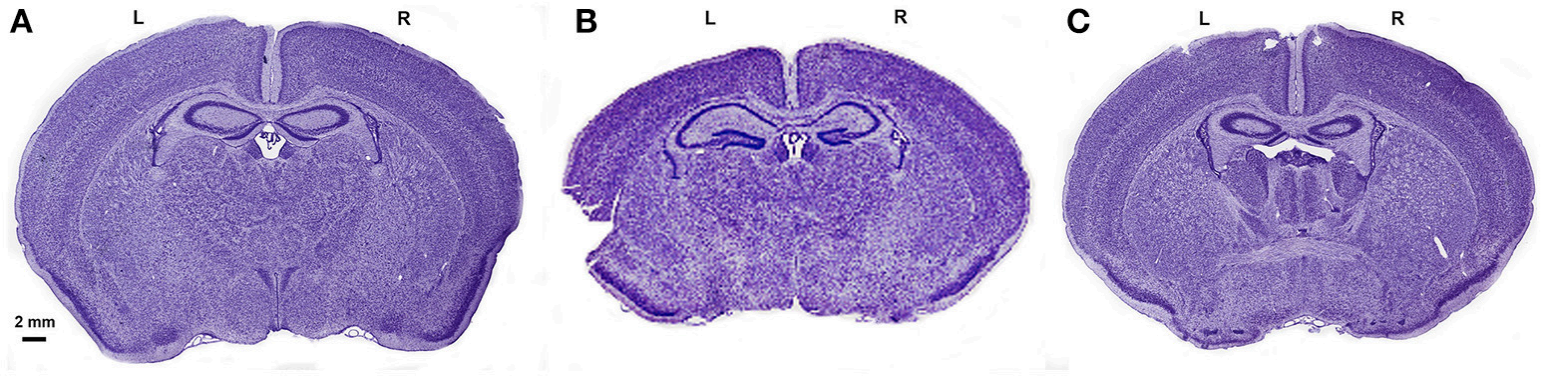
Cresyl violet staining of the brains from the 3 groups. Cresyl violet staining of the entire brain in a sham **(A)**, HI+A **(B)** and HI+CO **(C)**. The left side of the brain, L. The right side of the brain, R. The scale bar represents 2 mm.

At 12 days of age (5 days post injury), the ratio of ipsilateral to contralateral cortical volumes of HI+A (median 0.93, 25% 0.88, 75% 1.03, *n* = 10) was decreased compared to sham pups (median 1.01, 25% 0.97, 75% 1.25, *n* = 5) (*p* < 0.05). The HI+CO (treated with 1 h per day of 250 ppm of CO for 3 days post-injury) had significant preservation of the ratio of ipsilateral to contralateral cortex (median 1.07, 25% 0.97, 75% 1.23, *n* = 13) compared the HI+A group (*p* < 0.05) (Figures 3A and 4). All pups that were exposed to CO at 200 and 250 ppm survived the exposure.

### Hippocampal volumes

At 12 days of age (5 days post injury), similar to the cortical volumes, the ratio of ipsilateral to contralateral hippocampal volumes of the HI+A (median 0.76, 25% 0.52, 75% 0.84, *n* = 10) were decreased compared to the sham pups (median 1.0, 25% 0.88, 75% 1.14, *n* = 5) (*p* < 0.05). The HI+CO (treated with 1 h per day of 250 ppm of CO for 3 days post injury) preservation of the ratio of ipsilateral to contralateral hippocampus was not significantly different from the HI+A group (median 0.83, 25% 0.52, 75% 0.97, *n* = 13) (Figures 3B and 4).

### Weights, subjective testing and surface righting

The weights for the sham pups increased by 1.16 ± 0.17-fold from the first measure compared with an increase of 1.11 ± 0.18-fold in the HI+A group and 1.15 ± 0.21-fold in the HI+CO. There were no differences between the weights when the groups were compared (*p* > 0.05). The overall behavior of the pups were examined prior and after CO exposure, the CO exposed pups did not display any obvious behavioral differences compared to the Sham and HI+A group after CO exposure. All CO pups were subjectively observed to be Level 4 (alert, nursing well). In addition, during the CO exposure, the pups did not demonstrate any observable abnormal behavior or changes in behavior. The mean (±sd) time for righting was 1.41 ± 0.45 s in the sham pups (*n* = 17) compared with 1.22 ± 0.0.39 s in the HI+A pups (*n* = 19) and 1.18 ± 0.48 s in the HI+CO pups (*n* = 7). There were no differences between the groups (*p* > 0.05).

## Discussion

The major findings are that, using our preclinical established protocol, 250 ppm CO exposure (1) did not lead to a carboxyhemoglobin concentration that produce acute neurologic changes and (2) was effective in preserving cortical volumes following mild hypoxic-ischemic injury. This is the first report to demonstrate that CO given after HI in a neonatal model preserves cortical tissue. The data is promising and CO should therefore be investigated further as a potential synergistic therapy to be combined with therapeutic hypothermia.

CO is traditionally thought of as an environmental pollutant. Is it safe as a possible therapy in neonates? It is generally understood that inhalation of CO leads to its preferential binding with hemoglobin binding at 250 times greater affinity than oxygen (26). CO bound to hemoglobin produces carboxyhemoglobin which is a stable complex of CO and hemoglobin. In human adults, carboxyhemoglobin concentrations from 10 to 20% cause tightness across the forehead and possible headache, 20–30% cause headache, 30–40% cause severe headache, dizziness, and dim vision, 40–50% cause fainting, increased respiratory rate, and pulse, 50–60% cause coma with intermittent convulsions; 60–70% cause depressed cardiac function and depressed respiratory effort, and greater than 70% cause death (27). The highest average carboxyhemoglobin concentrations obtained in our experiments were 22% for 200 ppm and 27% for 250 ppm. The highest single carboxyhemoglobin concentration for the 250 ppm group was 31%. The average carboxyhemoglobin concentrations would produce only a mild headache in human patients if the same concentrations were obtained. This is assuming an exact translation to human subjects. However, the hemoglobin affinity for CO varies in mammalian species; thus, 1 h of inhalation of 250 ppm will increase carboxyhemoglobin to 15–20% in adult rats and hamsters, 10–12% in pigs and only 6–8% in healthy adult human subjects (28). These differences in carboxyhemoglobin among species relate primarily to the higher ventilation rates in smaller mammals (28). The concentration of 250 ppm of CO used for neuroprotection in our experimental design is also well below the reported fetal toxic threshold above 300 ppm of CO exposure when administered 24 h per day throughout pregnancy in mice (29). Fetal hemoglobin has a higher affinity for CO with a calculated ratio of 1.74 fetal vs. maternal % carboxyhemoglobin concentrations (29). Based on our results, CO, at 250 ppm in the neonatal mouse, produces carboxyhemoglobin concentrations within a non-toxic range. In addition to the monitoring of carboxyhemoglobin, we did not observe any changes in weight trends or behavior during or after exposure to CO. We performed a righting reflex which did not reveal differences between the groups. Given the mild injury, this test may not be sensitive enough to detect subtle differences between groups and we are currently performing long-term functional outcomes. It should be noted that the CO exposed group did not have a decrease in weight or a worsening performance compared to the other groups indicating that the treatment does not have a grossly negative impact on the pups.

The dose of 250 ppm was our target based on previous work from our laboratory demonstrating protection against transient focal ischemia in an adult mouse model. However, the design differed from our previous report in an adult mouse model of transient focal cerebral ischemia in which the 250 ppm was administered over 18 h (16). CO was administered for 1 h per day over 3 days. The design was based on a recent study in which CO was administered at 250 ppm for 1 h daily for 3 days prior to HI in a neonatal rat model (30). The study demonstrated a decrease in hippocampal apoptosis, an increase in the anti-apoptotic protein Bcl-2, and increased cytochrome c concentrations in CO-treated pups (30). Since the pups were exposed for 1 h and demonstrated an effect, we chose to emulate this design. Our study design differed in that we administered the CO post HI injury. This design was chosen to mimic the potential clinical scenario in which human neonates would be given the therapy post injury.

Similar to the work in adult mice with transient focal cerebral ischemia, there was preservation of cortical tissue in a neonatal mouse model of HI when given CO at 250 ppm (16). However, as noted above, the duration of administration was not as long—an 18-h single dose in the adult transient focal cerebral ischemia model vs. 1 h per day for 3 days in the neonatal HI model. The degree of cortical preservation was not as great as in the adult animal and this may relate to the dosing or the model. The injuries of the hypoxic-ischemic pups that were not treated with CO were not as significant as we expected. This may relate to the duration of exposure to hypoxia following the carotid artery ligation; the insult was relatively minor with only a 14% reduction in the cortical volume from the injury. It is, however, encouraging that CO could still preserve cortical volumes in the neonate following a minor HI insult. The finding is of clinical interest since neonates with mild HIE have been excluded from hypothermia trials due to earlier studies which showed that these neonates did not have long-term handicaps (defined as cerebral palsy, hearing or visual deficits, epilepsy or a score 3 SD below the population mean on the Stanford-Binet IQ test (9)). However, emerging data has shown that neonates with mild HIE may be at risk for brain injury. In a recent study, 50 neonates with mild HIE and who were not cooled underwent an MRI at 10 days of life (31). The MRI revealed injury in 40% of this mild group including near total injury in the basal ganglia and watershed areas of the cortex in 25% (31). Another study examined 104 neonates with a perinatal acidemia. These babies underwent a neurologic exam and 60 were found to have a mild encephalopathy. Of these 60, 12 (20%) experienced an abnormal short-term outcome (i.e., abnormal brain MRI, seizures, abnormal neurologic exam at discharge, need for gastronomy tube, death). Murray et al., in a prospective cohort study, examined the outcome of neonates with mild HIE at 5 years of age (32). Infants with mild HIE had significantly lower full, verbal, and performance IQs when compared to healthy control infants (32). Our data would suggest that CO may have therapeutic benefit in mild HIE.

Future experiments will expose the pups to longer durations of hypoxia post ligation to test if CO can have more of a significant impact on cortical volume preservation. Alternatively, the duration of exposure to CO may have to be increased.

The mechanism by which CO produces neuroprotection in the neonate following HI is currently unknown. CO protects against various insults similar to the pathophysiology of HIE through activation of anti-inflammatory, anti-apoptotic, and vasodilatory mechanisms (33–35). Our group has demonstrated that CO may act through the ARE-Nrf2-Keap1 pathway (17). This pathway is instrumental in regulating environmental stress by activating genes for antioxidants and detoxification. The pathway protects cells against inflammation. In its non-activated state, Nrf2 is bound to Keap1; following exposure to stressors the Nrf2-Keap1 complex dissociates and Nrf2 moves to the nucleus. Activation of this pathway may lead to many neuroprotective downstream mechanisms. In our experiments, the concentrations of carboxyhemoglobin decreased rapidly after administration of CO for 1 h, reaching concentrations comparable to controls after 60 min. Although speculative, the limited CO exposure could have activated many downstream pathways which led to preservation of cortical tissue by limiting inflammation and oxidative stress produced following hypoxia-ischemia in neonates, and this is actively being pursued using our preclinical model. Using a similar strategy in the clinical arena could minimize side effects from CO while producing beneficial downstream neuroprotective cascades.

A limitation of our study was the inability to sample carboxyhemoglobin from the same pup over time. The size of the pups precluded multiple samplings and the pups were sacrificed at each time point that the carboxyhemoglobin concentration was measured to obtain adequate blood volume for analysis. In addition, the cardiorespiratory status was also not monitored during the administration of CO and the neurologic outcomes were preliminary. Future studies will address any physiologic disturbances during the administration of CO and perform intricate neurologic testing to understand the short and long-term outcomes following CO exposure.

In summary, we have demonstrated that exogenous administration of CO did not produce carboxyhemoglobin concentrations which are associated with detectable CNS dysfunction and preserves cortical volumes following neonatal HI. Future experiments will focus on the functional outcomes of pups exposed to CO following HI, examining possible synergy with hypothermia and using transgenic mice to dissect the mechanism of action of CO in modulating injury post-HI through the ARE-Nrf2-Keap1 pathway. If future experiments are promising, large animal models will need to be utilized before proceeding with human trials, as rodents do not have hemoglobin F (only embryonic hemoglobin that switches to adult hemoglobin at E17). Thus, the pharmacokinetic of carbon monoxide in neonates should be tested in large mammals to adjust for fetal hemoglobin prior to human application.

## Author contributions

MD-E, MW, and SD designed, provided funding and expertise for, and analyzed all experiments, wrote the manuscript, and trained all staff in performing behavioral and histochemical experiments and data analyses; CR and AA trained and assisted in behavioral and histochemical experiments and analyses and edited the manuscript. CR and MM performed the behavioral analyses and quantified the histology. All authors have accepted the final version of the manuscript.

### Conflict of interest statement

The authors declare that the research was conducted in the absence of any commercial or financial relationships that could be construed as a potential conflict of interest.

## Abbreviations

CO: carbon monoxide
HI: hypoxia-ischemia.
